# Different expression domains for two closely related amphibian TAARs generate a bimodal distribution similar to neuronal responses to amine odors

**DOI:** 10.1038/srep13935

**Published:** 2015-09-11

**Authors:** Adnan S. Syed, Alfredo Sansone, Sebastian Röner, Shahrzad Bozorg Nia, Ivan Manzini, Sigrun I. Korsching

**Affiliations:** 1 Institute of Genetics, University of Cologne, Cologne, Germany; 2 Institute of Neurophysiology and Cellular Biophysics, University of Göttingen, Göttingen, Germany; 3 Center for Nanoscale Microscopy and Molecular Physiology of the Brain (CNMPB), University of Göttingen, Göttingen, Germany.

## Abstract

Olfactory perception is mediated by a multitude of olfactory receptors, whose expression in the sensory surface, the olfactory epithelium, is spatially regulated. A common theme is the segregation of different olfactory receptors in different expression domains, which in turn leads to corresponding segregation in the neuronal responses to different odor groups. The amphibian olfactory receptor gene family of trace amine associated receptors, in short TAARs, is exceedingly small and allows a comprehensive analysis of spatial expression patterns, as well as a comparison with neuronal responses to the expected ligands for this receptor family, amines. Here we report that TAAR4b exhibits a spatial expression pattern characteristically different in two dimensions from that of TAAR4a, its close homolog. Together, these two genes result in a bimodal distribution resembling that of amine responses as visualized by calcium imaging. A stringent quantitative analysis suggests the involvement of additional olfactory receptors in amphibian responses to amine odors.

Amphibians possess both a main and an accessory olfactory system, similar to mammals. However, the amphibian main olfactory epithelium (MOE) still contains a large number of microvillous sensory neurons, in addition to ciliated sensory neurons^1^. These two sensory neuron populations are to some extent segregated within the MOE^2^,^3^,^4^, resulting in corresponding inhomogeneities of the olfactory receptors, since the olfactory receptor gene families expressed in microvillous neurons are different from those found in ciliated neurons^2^,^5^. Neuronal responses to different odorants may be expected to reflect such inhomogeneities. Indeed, responses to amino acids are preferentially localized in the basolateral region of the MOE (microvillous neurons, lateral stream), whereas responses to alcohols, aldehydes and ketones are enriched in the apical and medial region of the MOE (ciliated neurons, medial stream), see^6^.

Lateral and medial stream segregate more sharply in the olfactory bulb. Amine-responsive glomeruli can be found in both the lateral and the medial olfactory bulb^6^, suggesting that amine responses may be bimodal, and carried by both odor-processing streams. Due to the sparse occurrence of amine-responding cells their spatial distribution in the amphibian olfactory epithelium has not been investigated yet.

Candidates for amphibian amine receptors would be trace amine-associated receptors (TAARs), since mammalian and fish TAARs have been shown to respond sensitively and specifically to amines^7^,^8^,^9^. *Taar* genes show large evolutionary dynamics resulting in repertoires of over one hundred genes in some ray-finned vertebrate species, e.g. zebrafish, whereas much smaller repertoires are found in the lobe-finned lineage (tetrapods, see^10^). In amphibians such as *Xenopus tropicalis* only a minute TAAR family of three genes was reported^10^, one of which (TAAR1) is not even expressed in the olfactory epithelium of *Xenopus*^6^ and other species^7^,^10^. We wished to investigate how such a small gene repertoire becomes integrated into spatial expression patterns dominated by much larger gene families such as odorant receptors (ORs^2^,^6^) and vomeronasal type-2 receptors (V2Rs^2^). We report here that the two *Xenopus taar* genes expressed in olfactory tissue, TAAR4a and TAAR4b, show two-dimensional expression patterns clearly distinct from each other and from those of OR and V2R receptors. TAAR4b occupies a basal expression zone, which is homogenous in the lateral-to-medial dimension, in contrast to the lateral-enriched and preferentially apical expression pattern of TAAR4a. Furthermore we show that amine-responsive cells follow a bimodal spatial pattern that could be partially explained by the spatial expression pattern of *taar4a* and *taar4b* genes. Our results are consistent with *taar* genes possessing their own unique expression zones, and possibly mediating amine responses, but also point to the existence of additional amine receptors in amphibian olfaction.

## Results

### Phylogenetic analysis identifies two additional *Xenopus tropicalis taar* genes

**Figure 1:**
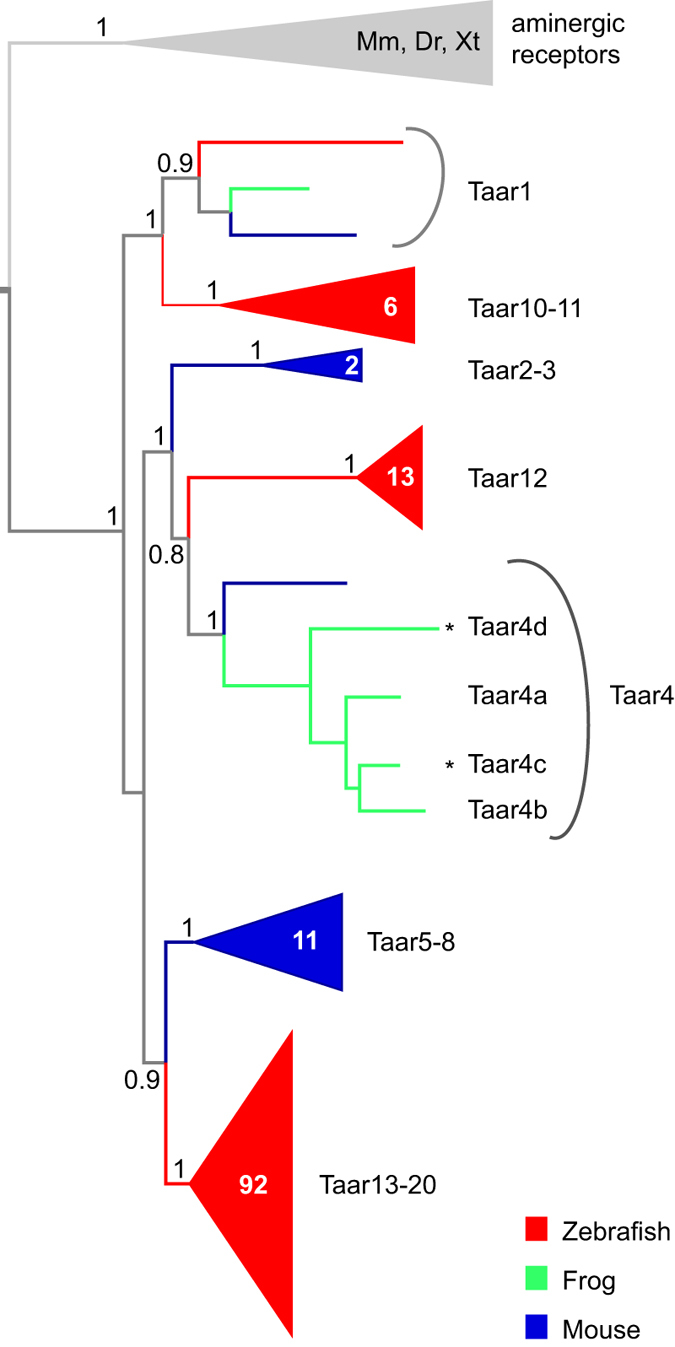
A single subfamily of olfactory taar genes in Xenopus tropicalis. Phylogenetic tree of *taar* gene repertoires of mouse, zebrafish and frog (*Xenopus tropicalis*), using aminergic neurotransmitter receptors as outgroup (grey). Tree branches for the different species are color-coded, red for zebrafish, blue for mouse, and green for frog. The tree was constructed using a modified Maximum Likelihood method (PhyML-aLRT). Some nodes are shown collapsed (triangle), the number of genes in these nodes is given inside the respective triangle. Branch support of selected nodes is shown as p values (1 equals p > 0.995). Sequences were taken from^10^ (*Danio rerio*, zebrafish; *Mus musculus*, house mouse; *Xenopus tropicalis*, Western clawed-frog; aminergic receptors as outgroup) or newly identified in databank searches (2 additional *Xenopus tropicalis* genes, asterisks).

The *taar* gene family of *Xenopus tropicalis* has been predicted to consist of just 3 genes, TAAR1, TAAR4a and TAAR4b^10^. To search for potential new additions to the databanks we have performed a thorough bioinformatic analysis using the Ensembl genome browser (http://www.ensembl.org/Xenopus_tropicalis/Info/Index), and the NCBI genomic databank (http://www.ncbi.nlm.nih.gov/assembly/GCF_000004195.2). *Taar* genes from several species (teleost fish as well as mammals) were used as queries. We found two additional members of the TAAR family that show the expected sequence motifs, including the TAAR fingerprint motif^11^ and that group with the established *taar* genes in the phylogenetic analysis (Fig. 1). Both genes belong to the TAAR4 subfamily, and were therefore named TAAR4c and TAAR4d. We have, however, not been able to clone orthologs of TAAR4c and TAAR4d from *Xenopus laevis*, unlike the previously described *taar* genes TAAR1, TAAR4a, and TAAR4b, for which *Xenopus laevis* orthologs were obtained by PCR-based homology cloning without difficulties. While we cannot exclude technical difficulties, it is also possible that gene losses have occurred in the *Xenopus laevis* TAAR4 subfamily compared to *Xenopus tropicalis.* A gene gain restricted to the *Xenopus tropicalis* lineage appears less likely, due to the considerable divergence within the TAAR4 subfamily (60–75% identity for pairwise comparisons).

### Two closely related *taar* genes exhibit very different expression zones

In previous experiments^2^,^4^,^6^ we had identified two axes, which together describe the spatial distribution of neurons expressing particular olfactory receptor genes. These are laminar height (apical-to-basal dimension) and perpendicular to this axis the position in the medial-to-lateral dimension. We found for several olfactory receptor genes that their distribution is broad, but not random, with preferred positions in both dimensions^2^,^6^, as observed previously for teleosts^12^.

**Figure 2:**
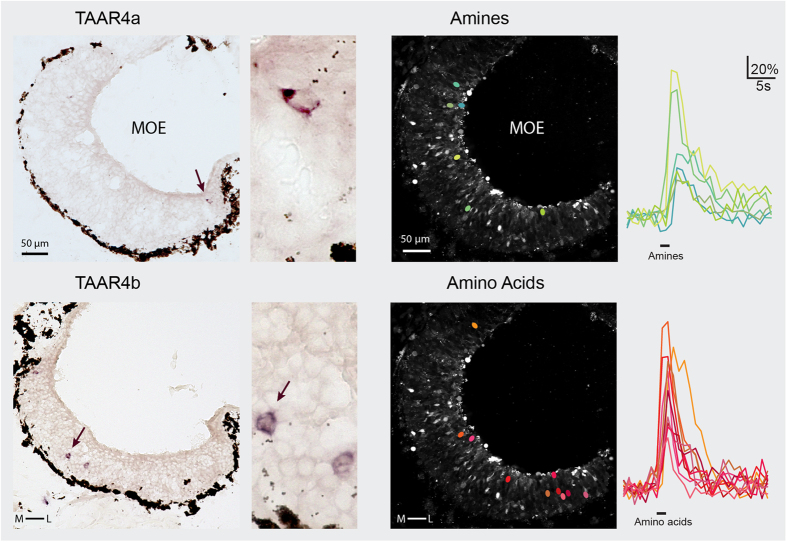
TAAR4a, 4b expression and amine responses occur in sparsely distributed olfactory neurons. *In situ* hybridization was performed for TAAR4a and TAAR4b using horizontal sections of larval *Xenopus laevis* head tissue. Enlargements containing labeled cell(s) are shown to the right of each complete section. A ring of dark brown melanophores delineates the basal border of the epithelium; apical is toward the lumen. TAAR4a-expressing cells are found apically and laterally, whereas TAAR4b-expressing cells lie more basal and show no obvious enrichment in the medial-to-lateral dimension. Amine- and amino acid-responsive cells were identified by calcium imaging (green and red ovals, respectively). Traces for all cells depicted by colored ovals are shown to the right of the respective micrographs, with odor stimulus period indicated by the black bar (fluorescence intensity values are given as ΔF/F, see Materials and Methods). To be able to show several traces for amines, a slice with above average frequency of amine-responsive cells was chosen.

**Figure 3:**
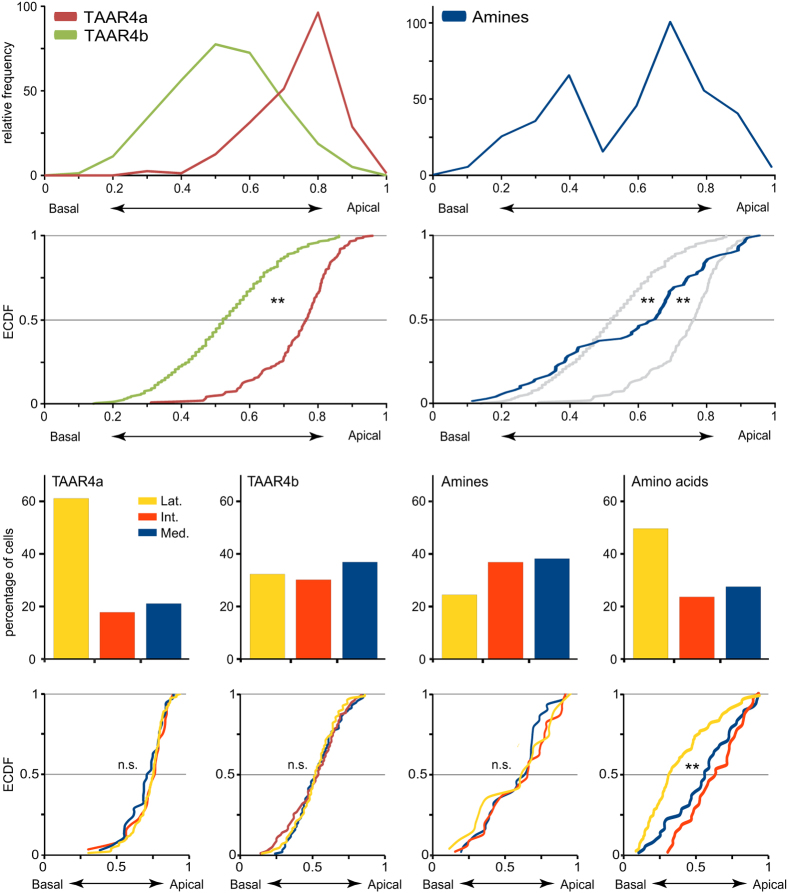
Quantitative analysis of spatial patterns. Basal-to-apical distributions were quantified for olfactory receptor genes and odor responses. Top row, receptor gene (left panel) and odor response (right panel) distributions are shown as histogram of relative height (0, most basal; 1, most apical position; bin size 0.1, bin center is shown). Left panel, *taar4a* (brown) is centered apical; *taar4b* (green) shows a basal peak. Right panel, amine responses (blue) show a bimodal distribution. Second row, distributions are depicted as empirical cumulative distribution function (ECDF). Color code as above, for comparison to the amine responses the receptor distributions are shown in grey in the right panel. **p < 0.01. Third row, medial-to-lateral distribution is shown as bar graph for TAAR4a, TAAR4b, amine responses, and amino acid responses (values taken from^2^ for comparison). Bottom row, basal-to-apical distributions are shown independently for each segment of the medial-to-lateral distribution depicted above. Medial segment, blue; intermediate segment, red; lateral segment yellow. Note that for both *taar* genes and amine responses the height distribution is undistinguishable between segments, in contrast to the significant difference between lateral and non-lateral amino acid responses (right panel). **p < 0.01.

**Figure 4:**
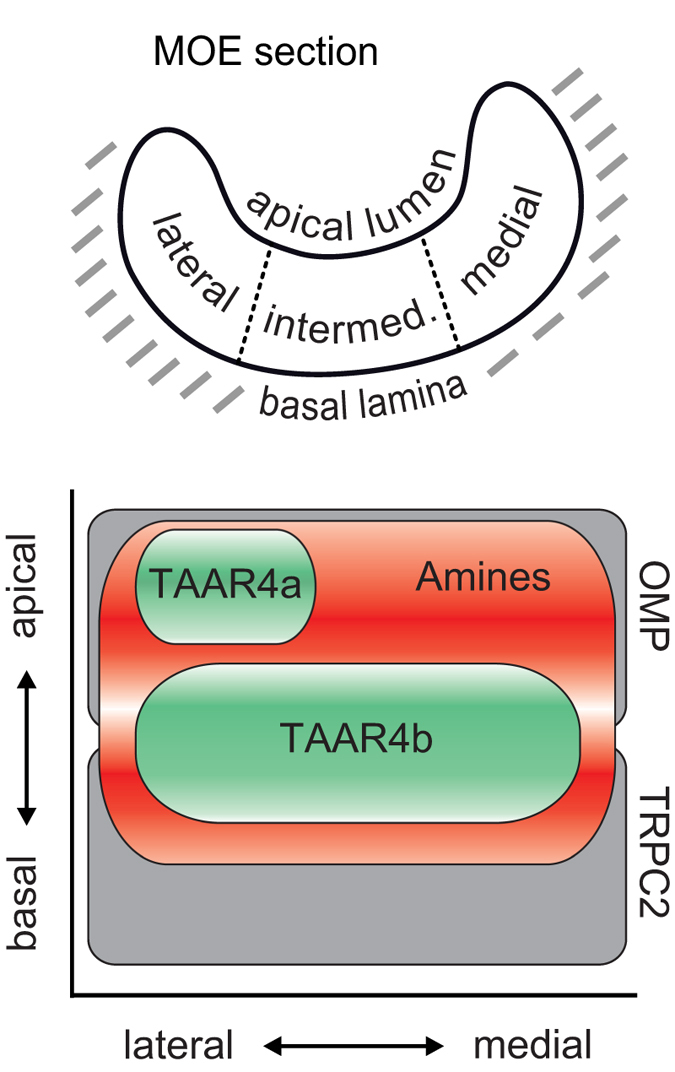
Schematic representation of TAAR4a,4b expression and amine responses. The top panel shows a horizontal section of the MOE of *Xenopus laevis* as it appears both for measuring odor responses and receptor gene expression. The basalmost layer of the epithelium borders the basal lamina, and the apicalmost layer borders the lumen. The apicalmost layer consists of supporting cells, and the basalmost region of proliferating cells, sandwiching the neuronal region. Perpendicular to this basal-to-apical coordinate, cells in the MOE can also be distinguished by position in the lateral, intermediate, or medial compartment (lateral-to-medial coordinate). The bottom panel depicts a schematic representation of the different cellular distributions analysed. Note that only the neuronal region of the MOE is shown here. Distributions are shown along two axes, basal-to-apical and lateral-to-medial. OMP and TRPC2 distributions are taken from earlier work of our groups^2^,^4^, and are shown for comparison. We emphasize that TAAR4a and TAAR4b differ in both dimensions, and together comprise a large subset of the bimodal amine response region.

Here we analysed the spatial patterns of TAAR4a and TAAR4b-expressing olfactory neurons by quantitative *in situ* hybridization in serial sections of the MOE in larval *Xenopus laevis*. Both genes show sparse expression as expected for olfactory receptor genes (Fig. 2). For TAAR4a-expressing neurons we find an apical and lateral-enriched distribution (Figs 3 and 4), consistent with previous observations^2^,^6^. However, the closely related TAAR4b shows a very different distribution. No enrichment is seen in the medial-to-lateral dimension, and in the apical-to-basal dimension the distribution of TAAR4b is centered more basally (Figs 3 and 4, Table 1). The differences between TAAR4a and 4b distributions are significant in both dimensions, as analysed by chi square test (p < 10^−7^, medial-to-lateral dimension) and Kolmogorov-Smirnov test (p < 0.001, laminar height) Table 1. Moreover, TAAR4b is expressed somewhat more frequently than TAAR4a, with 1.21 ± 0.08 cells/section compared to 0.27 ± 0.04 cells/section for TAAR4a (mean +/−SEM, n = 139 and 238 respectively; sections devoid of cells are included for calculation of the average). These frequencies are in the range observed for other single genes^2^.

### Amine responses are not lateralized but show a bimodal height distribution

Rodent TAAR receptors appear to be specialized for detection of various amines^7^,^8^, and recently a zebrafish TAAR receptor has also been shown to respond sensitively to some amines^9^. It is therefore conceivable that detection of amines constitutes an evolutionary conserved feature within the TAAR family. Indeed, amine responses have been shown for *Xenopus laevis* tadpoles^6^,^13^, but their underlying receptors are not known. If amine responses were mediated by TAARs, we would expect corresponding spatial patterns.

Here we performed a spatial analysis of amine responses in the MOE of *Xenopus laevis* tadpoles. Odor-induced neuronal activity was measured as intracellular calcium increase in acute slices of the MOE (Fig. 2), using a confocal microscope and fluo-4 as calcium indicator. A mix of thirteen different amines, among them the ligand for mouse TAAR4^8^,^14^, phenylethylamine, was used as odor stimulus. Amine-responding cells were sparse, 3.4 +/− 0.6 cells/section (mean +/−SEM, n = 23), confirming and extending previous observations^6^. In comparison, amino acid responses were over twofold more frequent, 8.1 +/− 1.3 cells/section (mean +/−SEM, n = 22) Thus, amine responses can be considered to result from a minor neuronal population within the epithelium, i.e. from expression of a few different receptors.

In the medial-to-lateral dimension amine responses showed no pronounced enrichment or depletion, in contrast to amino acid responses which show lateral enrichment (Figs 2, 3, 4). However, in the second dimension analysed, laminar height (basal-to-apical), we observed an unexpected bimodal distribution of amine-responding cells. Two frequency peaks at 0.4 and 0.7 relative height are separated by an intervening region with very few amine responses (Figs 3 and 4, Table 1). The two peaks in the histogram do not depend on the exact size and position of bins used, and are also visible (as plateau) in the empirical cumulative distribution function (ECDF) that does not involve binning (Fig. 3). Such a bimodal distribution suggests a heterogenous composition of the population of amine-responding cells. We would like to emphasize, that no difference in height distribution was observed between the three different compartments in the medial-to-lateral dimension (Fig. 3, Table 1), in contrast to the distribution observed for amino acid responses, where the lateral segment shows a basal peak and the medial segment an apical peak (Fig. 3), see also^2^. Thus, preferred positions of amine-responding cells appear to be specified independently for each dimension (apical-to-basal and medial-to-lateral).

### Partial overlap of TAAR expression zones with amine-responses

Remarkably, the apical peak of the height distribution of amine-responsive cells overlaps largely with the height distribution of TAAR4a-expressing cells, whereas the position of the basal peak is similar to that of TAAR4b-expressing cells (Fig. 3). In both cases the odor response peak is slightly shifted towards more basal positions compared to the peak seen for TAAR-expressing receptor neurons. Using identical assay methods^2^ we observed previously that the response to an agonist for ciliated neurons was likewise shifted towards slightly more basal positions, compared to the peak seen for a marker of ciliated neurons. In this case it seems clear that the slight difference in peak position ought to be due to technical reasons. Thus we conclude that the apparent small shift between the peak of TAAR-expressing neurons and of amine responses is most likely due to the difference in experimental conditions for both methods.

Both TAAR4b expression and amine-responsive cells show a homogenous distribution in the second dimension, medial-to-lateral. In other words, TAAR4b and the basal partition of amine-responsive cells overlap in both dimensions, consistent with the hypothesis that TAAR4b could mediate (some) amine responses in this region. In contrast, TAAR4a expression is centered in the lateral region, and consequently TAAR4a expression covers only a subregion of the apical partition of amine-responsive cells. Thus TAAR4a may carry lateral-and-apical amine responses, but for medial-and-apical amine responses additional receptors have to be postulated (Fig. 4).

In comparison to two large response domains described previously^2^,^6^, the height peak of the TAAR4a domain and the apical peak of the amine responses are identical to those observed for OMP and forskolin, respectively, both designating the ciliated neuron domain, whereas the height peak for the TAAR4b domain lies in between the ciliated neuron domain and the microvillous neuron domain as defined by TRPC2 expression^2^,^4^,^6^.

## Discussion

Amines are one of the main odor groups described for amphibians, and have been shown to mediate aversive reactions in mammals^14^,^15^ and fishes^9^. In these species olfactory receptors of the TAAR family have been deorphanized as amine receptors^7^,^9^. The amphibian TAAR family has been reported to consist of only three genes (one of which is not expressed in olfactory tissue^6^), allowing a comprehensive study of expression patterns and a thorough quantitative comparison with the spatial pattern of amine-responsive cells.

We report here that unexpectedly the spatial expression patterns of the remaining two receptors, the closely related TAAR4a and TAAR4b, are very different, both in terms of the height distribution and with respect to medial/lateral enrichment. TAAR4a is expressed apical and lateral, whereas TAAR4b is expressed basal and homogenous in the medial-to-lateral dimension. Thus, two distinctly different expression zones have resulted within the TAAR4 subfamily. Both zones are situated within the neuronal region of the *Xenopus* MOE, which is sandwiched between a thin layer of supporting cells contacting the lumen, and a basal layer of proliferating cells and immature neurons^16^.

The TAAR4b pattern is intercalated with respect to height between the spatial expression patterns of MOE-specific *v2r* genes (basal centered) and of *or* genes (apical centered), but shares a homogenous distribution in the medal-to-lateral dimension with OMP, a marker for ciliated neurons (note that another marker, G_olf_ is laterally depleted) and TRPC2, a marker for microvillous neurons^2^,^4^,^6^, see also (Fig. 4). Since TAAR4b-expressing cells are a minor cell population, they could conceivably be a constituent part of either cell type. However, both mammalian and zebrafish TAARs are known to be expressed in ciliated neurons^17^,^18^ (and unpublished observation, respectively), suggesting that this feature might be conserved in amphibians.

TAAR4a-expressing cells share their height distribution with ciliated neurons, suggesting an expression in ciliated neurons, but their distinct lateral enrichment in the medial-to-lateral dimension (Fig. 4) is not reflected in the distributions for ciliated marker genes (see above). However, ciliated neurons are present all along the medial-to-lateral dimension (including the lateral segment), and TAAR4a-expressing cells are an even smaller population than TAAR4b-positive cells, so their distribution would not influence the distribution of all ciliated neurons to any measurable degree. Thus, these data are consistent with TAAR4a being expressed in ciliated neurons.

Expression zones for both receptors together cover a large proportion of the amine-response region. Furthermore, amine-responding cells are rare, about 3 cells/explant, consistent with few receptors carrying the amine response. Conceivably, TAAR4a and 4b could mediate a considerable portion of the total amine response in *Xenopus laevis*. Together these receptors amount to about 1.5 cells/section. However, a direct comparison with the frequency of amine-responding cells is not possible due to the different assay paradigms. In any case, additional amine receptors have to be postulated to explain the apical and medial segment of amine responses, since this region is spared in the TAAR expression patterns analysed here. These receptors might be the putative orthologs of *Xenopus tropicalis* TAAR4c and 4d, which we were unable to clone, or may be found among both classes of ORs, which show an apical position and varying degrees of lateral depletion^6^ – such patterns would complement the laterally enriched TAAR4a. Unfortunately, a direct test of this hypothesis is beyond the scope of the present investigation, since the OR family comprises several hundred genes in *Xenopus*.

Taken together, we have shown that two closely related TAAR receptors exhibit expression zones distinctly different in two dimensions. Together these receptors cover a major proportion of the bimodal amine-responsive region, consistent with at least some of the amine responses being mediated by the *Xenopus laevis* TAAR4a and TAAR4b.

## Materials and Methods

### Sequence data mining and phylogenetic analysis

Using *Xenopus tropicalis* TAAR4a and TAAR4b, as well as representative TAAR sequences from two teleost fishes (zebrafish, medaka) and three mammals (opossum, cow, and mouse) as queries, we searched with tblastn (https://blast.ncbi.nlm.nih.gov/Blast.cgi) for *taar* genes in the *Xenopus tropicalis* genome version 4.1 (Joint Genome Institute, JGI, http://genome.jgi-psf.org/Xentr4/Xentr4.info.html). Homology regions above 200 amino acid length were considered further. Candidate sequences had to be located inside the phylogenetic tree with branch support over 80%, had to map to a unique, non-overlapping genomic position, and had to contain the TAAR fingerprint motif^11^ characteristic for that gene family. Two new sequences fulfilled all criteria, and were named according to phylogenetic relationship as TAAR4c and TAAR4d.

For the phylogenetic analysis published mouse and zebrafish TAAR sequences^10^ were included as ingroup, and several aminergic receptors as outgroup. The complete set of sequences was aligned using MAFFT, version 6, and E-INS-i strategy with default parameters^19^. The tree was constructed using a Maximum likelihood algorithm (PhyML-aLRT) with SPR setting for tree optimization and chi square-based aLRT for branch support^20^, as implemented on the phylemon2 server^21^. Figtree (http://tree.bio.ed.ac.uk/software/figtree/) was used for visualization of the phylogenetic tree. Branch support above 80% was considered significant.

### Animal Handling

All procedures for animal handling were approved by the governmental animal care and use office (Niedersächsisches Landesamt für Verbraucherschutz und Lebensmittelsicherheit, Oldenburg, Germany, Protocol No. T24.07) and were in accordance with the German Animal Welfare Act as well as with the guidelines of the Göttingen University Committee for Ethics in Animal Experimentation. Larval *Xenopus laevis*, stages 50–54, staged after^22^, were cooled to produce complete immobility and killed by transection of the brain at its transition to the spinal cord.

### Cloning and *In Situ* Hybridization

Nonambiguous primers for TAAR4b were designed based on published sequence information of homologous sequences in *Xenopus tropicalis*, taking care to avoid regions of possible cross-reactivity with TAAR4a^6^. An annealing temperature of 56 °C was used with genomic DNA as template, and a fragment with the predicted length of 516 bp was obtained, cloned into pGEM-T (Promega, Mannheim, Germany) and later confirmed by sequencing.

*In situ* hybridization was performed using digoxigenin-labeled (DIG; Roche Molecular Biochemicals, Mannheim, Germany) RNA probes prepared from the cloned DNA by using the same forward primers and reverse primers with a T3 promoter site attached to their 5′ end. For *in situ* hybridization, tissue blocks containing MOE and vomeronasal organ were cut horizontally, fixed in 4% (wt/vol) formaldehyde solution for 2 h at room temperature, equilibrated in 30% saccharose, and embedded in Jung tissue-freezing medium (Leica, Bensheim, Germany). Cryostat sections of 10–12 μm (Leica CM1900) were dried at 55 °C and postfixed in 4% (wt/vol) paraformaldehyde for 10–15 min at room temperature. Hybridizations were performed overnight at 60 °C in 50% (vol/vol) formamide using standard protocols. Anti-DIG primary antibody coupled to alkaline phosphatase and NBT-BCIP (4-nitro blue tetrazolium chloride, and 5-bromo-4-chloro-3-indolyl-phosphate, both from Roche Molecular Biochemicals) were used for signal detection.

### Calcium Imaging

Odor responses were measured as changes of intracellular calcium concentrations of individual olfactory neurons in vibratome slices of the olfactory organs using Fluo-4/AM as calcium indicator dye, essentially as described previously^2^,^23^. A mix of thirteen amines (2-Phenylethylamine, tyramine, butylamine, cyclohexylamine, hexylamine, 3-methylbutylamine, N,N-dimethylethylamine, 2-methylbutylamine, 1-formylpiperidine, 2-methylpiperidine, N-ethylcyclohexylamine, 1-ethylpiperidine, piperidine) was used to analyse amine responses, (*cf.*^6^). For comparison an L-amino acid mix (19 amino acids, *cf.*^6^) was used. Odorant stimuli were prepared at 100 μM concentration per compound and applied by gravity feed into a small carrier flow, i.e. final concentration was somewhat lower than 100 μM. This concentration was chosen in accordance with previous publications using the same assay system^2^,^3^,^6^ and has also been used for other aquatic species^24^. The minimum interstimulus interval was 2 minutes to avoid adaptation. Reproducibility of neuronal responses was verified by repeating the application of each stimulus at least twice. Fluorescence images of the whole MOE were acquired at 1 Hz (excitation at 488 nm; emission above 505 nm) using a laser-scanning confocal microscope (LSM 510/Axiovert 100 M; Zeiss, Jena, Germany) and analyzed using custom programs written in MATLAB (MathWorks, Natick, USA). Active olfactory neurons were identified as regions of high cross-correlation between the fluorescence signals of neighboring pixels, as described in^25^. The diameter of such regions was typically 6–10 μm, consistent with these signals emanating from the somata of individual olfactory neurons^26^. Optical section thickness was chosen to ensure that observed signals originated from single cells. Ten images before the onset of stimulus application were taken as control (F_0_). In accordance with previous publications^2^,^3^,^13^,^16^,^23^ a response was considered significant if three criteria were fulfilled: it had to occur within 10 s after stimulus application, the first two fluorescence values after stimulus arrival at the mucosa, F_1_ and F_2_, had to be larger than the maximum of the F_0_ values, and F_2_ had to be larger than F_1_.

### Analysis of Spatial Distribution

The position of cells was evaluated in two dimensions perpendicular to each other, medial-to-lateral and basal-to-apical. Position in the first dimension was determined according to^6^, and in the second dimension according to^2^. The relative height of the cell was defined as distance of the cell soma center from the basal border of the epithelium divided by total thickness of the epithelial layer at the position of the cell (h_rel_ = h_cell_/h_layer_). Cell positions were measured using ImageJ (http://rsbweb.nih.gov/ij/) and/or manually on printouts. Distributions are visualized as histograms with 10 bins (x value given corresponds to the bin center), or unbinned as empirical cumulative distribution function (ECDF). Median, skewness, and half-width of the spatial distributions were calculated from unbinned values using Open Office (version 3.2; www.openoffice.org/). Half-width of a height distribution was defined as difference between the height values for the upper quartile and the lower quartile. The peak value was taken from the graphical representation of the histograms. To estimate whether two height distributions were significantly different, we performed Kolmogorov–Smirnov tests on the unbinned distributions, see^27^. This test is particularly suitable for continuous distributions and makes no assumptions about the nature of the distributions investigated, which is essential because the observed distributions are not Gaussian. Due to the sensitive nature of the test for large distributions (n > 100), we selected P < 0.01 as cutoff criterion for significant difference.

## Additional Information

**How to cite this article**: Syed, A. S. *et al.* Different expression domains for two closely related amphibian TAARs generate a bimodal distribution similar to neuronal responses to amine odors. *Sci. Rep.* **5**, 13935; doi: 10.1038/srep13935 (2015).

## Figures and Tables

**Table 1:**
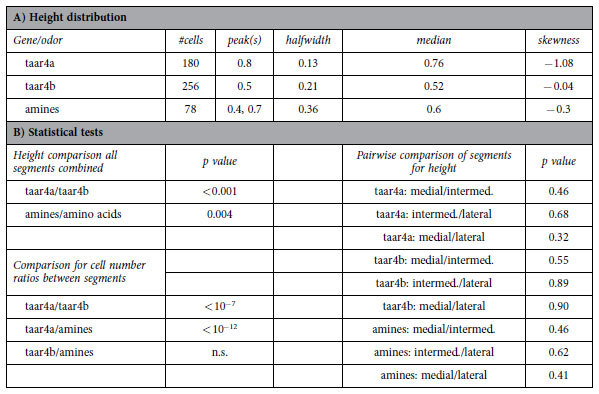
Statistical analysis of spatial distributions.

(A) Number of cells analysed and characteristic parameters for distributions shown in (Fig. 3). (B) Pairwise comparisons of different genes and/or odor responses were performed using the Kolmogorov–Smirnov test of the unbinned height distributions or chi square test (for comparing cell number ratios between lateral, intermediate, and medial segments). Values for amino acid-responsive cells were taken from^2^. Distributions were considered significantly different for p < 0.01.

## References

[b1] Hansen A., Reiss J. O., Gentry C. L. & Burd G. D. Ultrastructure of the olfactory organ in the clawed frog, *Xenopus laevis*, during larval development and metamorphosis. J Comp Neurol 398, 273–288 (1998). 9700571 10.1002/(sici)1096-9861(19980824)398:2<273::aid-cne8>3.0.co;2-y

[b2] Syed A. S., Sansone A., Nadler W., Manzini I. & Korsching S. I. Ancestral amphibian v2rs are expressed in the main olfactory epithelium. Proc Natl Acad Sci USA 110, 7714–7719 (2013). 23613591 10.1073/pnas.1302088110 PMC3651509

[b3] Sansone A., Hassenklöver T., Syed A. S., Korsching S. I. & Manzini I. Phospholipase C and diacylglycerol mediate olfactory responses to amino acids in the main olfactory epithelium of an amphibian. PLoS One 9, e87721 (2014). 24489954 10.1371/journal.pone.0087721 PMC3905040

[b4] Sansone A., Syed A. S., Tantalaki E., Korsching S. I. & Manzini I. Trpc2 is expressed in two olfactory subsystems, the main and the vomeronasal system of larval *Xenopus laevis*. J Exp Biol 217, 2235–2238 (2014). 24737764 10.1242/jeb.103465 PMC4986728

[b5] Buck L. B. Olfactory receptors and odor coding in mammals. Nutr Rev 62, S184–188; discussion S224–141 (2004). 15630933 10.1111/j.1753-4887.2004.tb00097.x

[b6] Gliem S. *et al.* Bimodal processing of olfactory information in an amphibian nose: odor responses segregate into a medial and a lateral stream. Cell Mol Life Sci 70, 1965–1984 (2013). 23269434 10.1007/s00018-012-1226-8 PMC3656224

[b7] Liberles S. D. & Buck L. B. A second class of chemosensory receptors in the olfactory epithelium. Nature 442, 645–650 (2006). 16878137 10.1038/nature05066

[b8] Zhang J., Pacifico R., Cawley D., Feinstein P. & Bozza T. Ultrasensitive detection of amines by a trace amine-associated receptor. J Neurosci 33, 3228–3239 (2013). 23407976 10.1523/JNEUROSCI.4299-12.2013 PMC3711460

[b9] Hussain A. *et al.* High-affinity olfactory receptor for the death-associated odor cadaverine. Proc Natl Acad Sci USA 110, 19579–19584 (2013). 24218586 10.1073/pnas.1318596110 PMC3845148

[b10] Hussain A., Saraiva L. R. & Korsching S. I. Positive Darwinian selection and the birth of an olfactory receptor clade in teleosts. Proc Natl Acad Sci USA 106, 4313–4318 (2009). 19237578 10.1073/pnas.0803229106 PMC2657432

[b11] Lindemann L. *et al.* Trace amine-associated receptors form structurally and functionally distinct subfamilies of novel G protein-coupled receptors. Genomics 85, 372–385 (2005). 15718104 10.1016/j.ygeno.2004.11.010

[b12] Weth F., Nadler W. & Korsching S. Nested expression domains for odorant receptors in zebrafish olfactory epithelium. Proc Natl Acad Sci USA 93, 13321–13326 (1996). 8917589 10.1073/pnas.93.23.13321 PMC24091

[b13] Gliem S., Schild D. & Manzini I. Highly specific responses to amine odorants of individual olfactory receptor neurons *in situ*. Eur J Neurosci 29, 2315–2326 (2009). 19490026 10.1111/j.1460-9568.2009.06778.x

[b14] Ferrero D. M. *et al.* Detection and avoidance of a carnivore odor by prey. Proc Natl Acad Sci USA 108, 11235–11240 (2011). 21690383 10.1073/pnas.1103317108 PMC3131382

[b15] Kobayakawa K. *et al.* Innate versus learned odour processing in the mouse olfactory bulb. Nature 450, 503–508 (2007). 17989651 10.1038/nature06281

[b16] Hassenklöver T. *et al.* Nucleotide-induced Ca^2+^ signaling in sustentacular supporting cells of the olfactory epithelium. Glia 56, 1614–1624 (2008). 18551628 10.1002/glia.20714

[b17] Dewan A., Pacifico R., Zhan R., Rinberg D. & Bozza T. Non-redundant coding of aversive odours in the main olfactory pathway. Nature 497, 486–489 (2013). 23624375 10.1038/nature12114 PMC3663888

[b18] Johnson M. A. *et al.* Neurons expressing trace amine-associated receptors project to discrete glomeruli and constitute an olfactory subsystem. Proc Natl Acad Sci USA 109, 13410–13415 (2012). 22837392 10.1073/pnas.1206724109 PMC3421222

[b19] Katoh K., Misawa K., Kuma K. & Miyata T. MAFFT: a novel method for rapid multiple sequence alignment based on fast Fourier transform. Nucleic Acids Res 30, 3059–3066 (2002). 12136088 10.1093/nar/gkf436 PMC135756

[b20] Guindon S. *et al.* New algorithms and methods to estimate maximum-likelihood phylogenies: assessing the performance of PhyML 3.0. Syst Biol 59, 307–321 (2010). 20525638 10.1093/sysbio/syq010

[b21] Sanchez R. *et al.* Phylemon 2.0: a suite of web-tools for molecular evolution, phylogenetics, phylogenomics and hypotheses testing. Nucleic Acids Res 39, W470–474 (2011). 21646336 10.1093/nar/gkr408 PMC3125789

[b22] Nieuwkoop P. D. & Faber J. (Eds): Normal Table of *Xenopus laevis* (Daudin). New York: Garland Publishing; (1994).

[b23] Hassenklöver T., Pallesen L. P., Schild D. & Manzini I. Amino acid- vs. peptide-odorants: responses of individual olfactory receptor neurons in an aquatic species. PLoS One 7, e53097 (2012). 23300867 10.1371/journal.pone.0053097 PMC3531423

[b24] Fuss S. H. & Korsching S. I. Odorant feature detection: activity mapping of structure response relationships in the zebrafish olfactory bulb. J Neurosci 21, 8396–8407 (2001). 11606628 10.1523/JNEUROSCI.21-21-08396.2001 PMC6762795

[b25] Junek S., Chen T. W., Alevra M. & Schild D. Activity correlation imaging: visualizing function and structure of neuronal populations. Biophys J 96, 3801–3809 (2009). 19413986 10.1016/j.bpj.2008.12.3962 PMC2711456

[b26] Manzini I. & Schild D. Classes and narrowing selectivity of olfactory receptor neurons of *Xenopus laevis* tadpoles. J Gen Physiol 123, 99–107 (2004). 14744986 10.1085/jgp.200308970 PMC2217426

[b27] Press W. H., Teukolsky S. A., Vetterling W. T. & Flannery B. P.: Numerical Recipes in C: The Art of Scientific Computing. Cambridge: Cambridge University Press (1992).

